# Rapidly Deployable IoT Architecture with Data Security: Implementation and Experimental Evaluation

**DOI:** 10.3390/s19112484

**Published:** 2019-05-31

**Authors:** Sudip Maitra, Kumar Yelamarthi

**Affiliations:** College of Science and Engineering, Central Michigan University, Mt Pleasant, MI 48859, USA; maitr1s@cmich.edu

**Keywords:** block ciphers, edge computing, IoT security, modular architecture, AES, XTEA

## Abstract

Internet of Things (IoT) has brought about a new horizon in the field of pervasive computing and integration of heterogeneous objects connected to the network. The broad nature of its applications requires a modular architecture that can be rapidly deployed. Alongside the increasing significance of data security, much research has focused on simulation-based encryption algorithms. Currently, there is a gap in the literature on identifying the effect of encryption algorithms on timing and energy consumption in IoT applications. This research addresses this gap by presenting the design, implementation, and practical evaluation of a rapidly deployable IoT architecture with embedded data security. Utilizing open-source off-the-shelf components and widely accepted encryption algorithms, this research presents a comparative study of Advanced Encryption Standards (AES) with and without hardware accelerators and an eXtended Tiny Encryption Algorithm (XTEA) to analyze the performance in memory, energy, and execution time. Experimental results from implementation in multiple IoT applications has shown that utilizing the AES algorithm with a hardware accelerator utilizes the least amount of energy and is ideal where timing is a major constraint, whereas the XTEA algorithm is ideal for resource constrained microcontrollers. Additionally, software implementation of AES on 8-bit PIC architecture required 6.36x more program memory than XTEA.

## 1. Introduction

Internet of Things (IoT) is a world-wide network of interconnected objects that are uniquely addressable, based on standard communication protocols [1]. The “Thing” in IoT can be anything from toasters, thermostats, wearable electronics, assistive devices, smart-vehicles, structural health monitoring systems, environmental monitoring, agriculture, smart homes, and industrial automation systems [2,3]. More devices are now connected with the internet, as pervasive computing proliferates at a blistering pace to reduce human intervention, increase ease of use and improve efficiency. As the technology pervades different facets of our lives, it also brings its own share of challenges as all these sensors and smart devices are constantly collecting and sharing data about our surroundings, inherently putting consumer data privacy and security at risk [4,5]. As the IoT system collects and exchanges sensitive, private data, ensuring security across all levels of the architecture is essential yet most IoT devices at the end nodes have significant security concerns. Typically, IoT devices are constrained with low memory, limited power, and computational capabilities to implement traditional encryption algorithms [6,7]. In resource constrained IoT architectures, it is necessary to employ a lightweight encryption to provide security to sensitive information while minimizing the overhead in memory, time [8] and power [9].

Implementation of an IoT system involves interconnecting a large number of devices from diverse manufacturers and industries, and performance varies by applications and user requirements, and therefore, heterogeneity of devices and information is an important consideration in a unified architecture for IoT. Accordingly, architecture is the backbone of an IoT system [10]. Furthermore, it is imperative to have a flexible layered architecture, especially at a time when the ever-increasing number of architectures have not yet converged to a reference model [11]. While numerous IoT architectures have been published, each has been customized to a respective application [12,13,14], with not much emphasis on modularity for replication in diverse application, and rapid deployment. Addressing these challenges, this research presents a modular architecture termed, Lab-in-a-Box (LiB), a lightweight, low-cost sensor platform for rapid deployment in diverse applications. It attempts to provide a solution by providing a versatile, flexible and modular architecture that can be easily modified depending on the application and can be rapidly deployed requiring minimum set-up time and effort. The core concept of the LiB is a perceptible modular IoT architecture that enables a wide range of application-specific layers to interact with other sub-layers of the whole structure seamlessly, reducing complications, while integrating the different sections of an IoT application.

The existing research provides many solutions for security in resource-constraint devices [15,16,17,18,19,20,21,22,23] and IoT architectures for specific applications [10,12,24]. However, no study has addressed these issues together and provided insight into how encryption affects the performance of an IoT system in terms of system memory, timing, and energy consumption. This paper attempts to tie in those issues into one work to investigate the effects and implications of encryption algorithms on IoT applications. This paper is organized as follows: Section 2 highlights current research on light-weight encryption algorithms and modular IoT architecture. Section 3 describes the LiB architecture and expands on widely accepted encryption algorithms. Section 4 expands on the proposed LiB architecture with data encryption for three different applications. Comparative results and discussions are presented in Section 5, followed by conclusions in Section 6.

## 2. Related Works

### 2.1. Encryption Algorithms

Encryption algorithms can be broadly classified into three different types, asymmetric encryption, symmetric encryption, and hash functions. Although asymmetric cryptographic algorithms are flexible, they require higher power, larger storage and consume more power, which renders them unsuitable for securing IoT applications. Symmetric cryptographic encryption includes stream and block ciphers, which are faster and use simple fast operations, making them suitable for IoT applications. Stream ciphers are fast but are suited for streaming data, whereas block ciphers are more flexible in terms of application. Several of such lightweight block ciphers have been proposed to secure resource constrained devices in IoT applications. Zhang et al. assessed various security schemes and power consumption by Wireless Sensor Network (WSN) protocols [25]. The WSN nodes feed the data to the base station (BS), where the data is processed, and relayed to the cloud if any further processing is required. Xiao et al. simulated the encryption and decryption (AES algorithm) overhead in microseconds [26]. They discussed routing protocols, characteristics, limitations, vulnerabilities, and countermeasures. Barahtian et al. implemented and evaluated block ciphers on 8-bit, 16-bit and 32-bit microcontrollers focusing on the widely used algorithm tinyAES and others suitable for embedded platforms [27].

Noura et al. [28] stated that traditional cryptographic algorithms use a static structure, which requires several rounds of computation, adding significant overhead on execution time and constraint on computational resources. They further stated that the problem is compounded for multimedia data because the algorithms have stringent quality of service requirements. They offered a dynamic cipher structure, which minimizes the rounds to a single one without compromising randomness and security. In [29], Biryukov et al. provided a list of algorithms and cryptanalysis against them. The author split lightweight cryptography into two groups namely, ultra-lightweight cryptography and ubiquitous cryptography. The authors provided design constraints that make an algorithm lightweight in terms of hardware and software. They also provided a benchmark for assessing good performance on a given platform.

AES is a widely-used block cipher for security in IoT applications such as IEEE802.15.4, LoRaWAN, Zigbee, Sigfox, ZWave, and has readily available hardware accelerators built into commercial products. When code size and Random Access Memory (RAM) space is not of concern, AES is a strong candidate for providing security to small devices [15,16,17,18]. However, AES implementation in software introduces time delay in data processing and transmission, and increases power and energy consumption. As such, researchers have custom designed AES hardware accelerators for implementation in portable IoT applications [30,31], that is often not feasible for rapid deployment of IoT applications. Another alternative is using reprogrammable Field Programmable Gate Arrays (FPGA), but requires additional modules such as data-converters that are often not built-in. Optimized serial architectures like 8-b architectures for hardware implementation of AES are suitable for constraint devices as they require less area and energy but yield lower throughput. Another approach to solving the issue of securing resource constrained devices in IoT is assessing the feasibility of ultra-lightweight block ciphers such as SIMON and SPECK [19], PRESENT [21], and CLEFIA [32]. SIMON is a versatile lightweight block cipher based on Feistal networks, tailored for hardware implementation in resource constrained devices. Different datapath architectures for SIMON have been proposed and studied, which focus on area [33,34], latency [34], throughput [31,34], energy efficiency [34], and security [34]. In other works, SIMON is shown to be promising in terms of area and throughput through simulations [23], but not much published literature exists to demonstrate its implementation in commodity hardware like microcontrollers manufactured by popular companies such as TI, Microchip, ST, etc. KATAN is a lightweight block cipher, but requires a large area to be implemented for IoT devices [20]. Light Encryption Device (LED) has an adjustable security level, but the performance is not on par with other algorithms such as PRESENT [15,21,22]. Although newer block ciphers like PRESENT and CLEFIA require less area and smaller memory, it comes at a cost of reduced security levels and low throughput and as a result, are not yet adopted in new IoT proposals. Moreover, software implementation of PRESENT is not as effective and is complex [23]. In [31], authors propose optimization strategies for AES 32-b architecture to maximize throughput, reduce area, power, and energy. Their proposed AES implementation consumes almost the same amount of energy as PRESENT, making it a suitable candidate for future ultra-low power IoT applications. Other lightweight algorithms such as SEA and TEA have similar performance, but TEA requires less code-space [23]. Among the lightweight algorithms, TEA matches the speed and conciseness of hardware accelerated AES more than any other encryption algorithm [15,22,23]. XTEA was developed to address the vulnerabilities that existed in TEA [35].

Overall, AES was found to be the most widely used, optimized for constrained hardware, and the byte-oriented operations work efficiently on 8-bit, 16-bit and 32-bit architectures. AES and XTEA lightweight encryption algorithms are employed to provide security to edge node devices in IoT applications. The modularity of these algorithms and compatibility with low resource hardware make them ideal candidates for research to find the feasibility of using them in applications where developers have to tune the trade-off between cost, light weight devices, and security.

In this paper, we address the security issues in IoT edge nodes. Although blockchain technology has been considered to provide security in IoT systems, it is more targeted at higher levels of the IoT architecture as it requires significant energy, introduces time delay and computational overhead [36,37]. While there are attempts to use blockchain technology for security at IoT edge nodes in simulation, this is currently beyond the scope of the current work, and will be addressed in the future [38].

### 2.2. IoT System Architecture

Monitoring of building occupancy and environment is essential for fault detection, intelligent control, and building commissioning. Although there are many solutions for environmental monitoring, they are time-consuming and require technical knowledge to set up. The three applications implemented in this paper represent environmental sensing solutions in different scenarios. These sensor platforms gather information from their surroundings, feed the data to the wireless transceivers through the first layer of the modular architecture discussed later in this section. Although some wireless transceivers were equipped with standard security protocols, not all transmit encrypted data. The raw data can be accessed between a wireless transceiver and gateway device, jeopardizing privacy and security. This paper adds an intermediate layer of security between the information collector node and wireless transceiver layer, which is nonexistent in most literature and consumer products so that raw data cannot be easily accessed remotely, even if the wireless transceiver does not have any encryption features.

This section briefly describes the state-of-the-art literature on such platforms to provide an insight into how researchers investigate the efficacy of such devices and tuning of parameters that ameliorate the performance and usability of sensor platforms. Building-in-Briefcase (BiB) [39] is a portable sensor network platform that addresses issues such as portability, accessibility, and scalability. The distributed sensor network collects environmental data, sends the data to a router using a TCP/IP protocol and Wi-Fi, the router sends the data to the central database over the internet using 3G connectivity. The data can be viewed on a web portal in real time. The BiB sensors are connected to the router using standard wireless 802.11 b/g technology secured with WPA2. The radio used to transfer the sensor data to the router wirelessly is Roving Networks’ RN-XV. It consumes 10x more power than Bluetooth or IEEE 802.15.4 products, but the high data rate allows for the transmission of large data packets in one burst, which they claim are more power efficient.

WE-Safe [40] is a real-time, low power wearable sensor node platform that supports multiple environmental sensors and is aimed at monitoring harmful gases and elements such as carbon monoxide, carbon dioxide, particulate matter, and ultraviolet rays. The sensor node uses the Long Range (LoRa) based gateway to establish communication between the environmental sensors and the cloud. In [41], the authors proposed a Raspberry Pi based portable tour guide system, which gives personalized, localized audio-visual information to tourists to improve user-experience and increase tour efficiency. This handheld portable tour guide system uses iBeacon for localization and RFID technology to detect specific objects such as artifacts in museums. The iBeacons are placed at specific locations and send advertising packets periodically containing its universally unique identifier (UUID). The Wi-Fi enabled Raspberry Pi in the tour guide device measures the received signal strength identifier (RSSI) of the iBeacons to find out the tourist’s location. The location of the tourists and the identity of the objects are uploaded to a cloud service, which sends uniform resource locators (URLs) of a video to the handheld tour guide device providing detailed information about the object. Gupta et al. presented a wireless environmental monitoring system for greenhouses in [42], where the system is divided into three functional units. The sensor unit and the coordinator units are C8051F020 microcontrollers and communicate with each other via Zigbee wireless technology.

**Figure 1:**
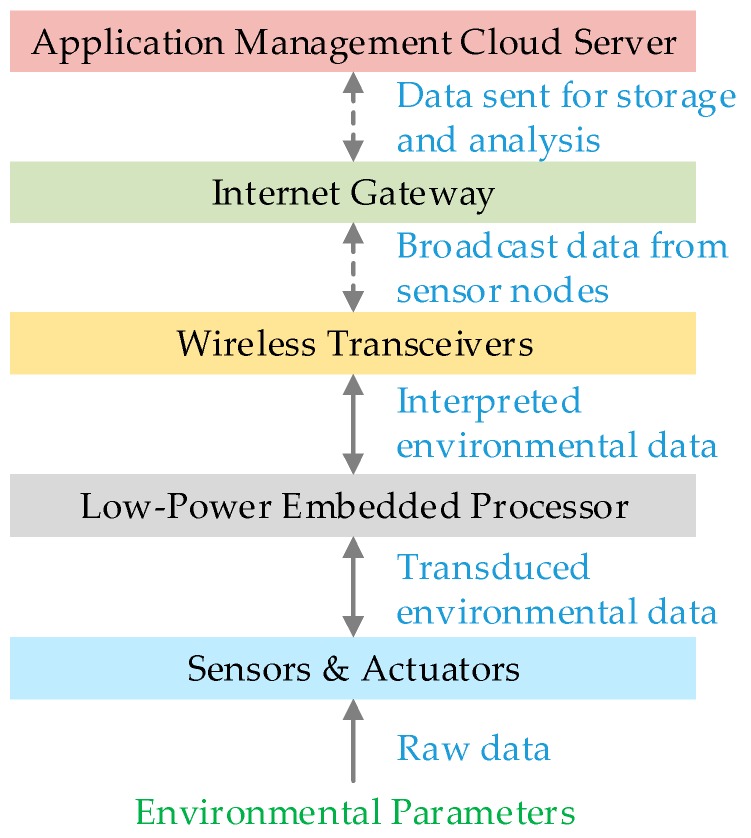
Architecture and information flow in an IoT system [10].

From these studies, it is evident that the breadth of IoT applications is broad, but the connectivity and overall system architecture is fundamentally similar even though the hardware utilized varies based on the application. This interconnection of heterogeneous objects requires ad-hoc networks, which most of the time are neither scalable nor interoperable. Guan et al. [43,44] proposed a top-down approach to developing an IoT application for rapid deployment. Their proposed TinyLink system enables application developers to write their hardware independent application code and generate a configuration for hardware platforms and hardware dependent binary code ready for porting. This can be thought of as an analog of optimizer function used in compilers for hardware. Software optimizers are great and useful but they do have limitations, and certain high-level optimization always requires human intervention. However, this can reduce the time and cost of developing a new application. This also provides an idea that the system architecture of IoT can be broken up into functional blocks and can be made modular with some high-level perspective and understanding of each layer as in Figure 1.

The first layer consists of sensors that collect environmental parameters and actuators that serve as feedback devices, and are aggregated by the embedded processor in the second layer. The third layer is the wireless transceiver module such as Bluetooth Low Energy (BLE), Wi-Fi, ZigBee, which provides wireless connectivity to the sensor nodes. Typically, these wireless devices create a type of mesh network for covering a large area. In some applications, the embedded hardware layer has an integrated network stack so the second and third layer is merged into one unit. While a few wireless transceivers follow specific protocols of communication and conform to security requirements, the majority do not. Therefore, the data is vulnerable when it is transiting from the wireless transceiver layer to the internet gateway layer. The fourth layer is the central internet gateway device, which sends the sensor data to the next layer, the cloud server via Wi-Fi, 3G/4G or Ethernet connectivity, where the data is stored for future analysis.

## 3. Lab-in-a-Box (LiB) Architecture

### 3.1. Modular System Architecture

**Figure 2:**
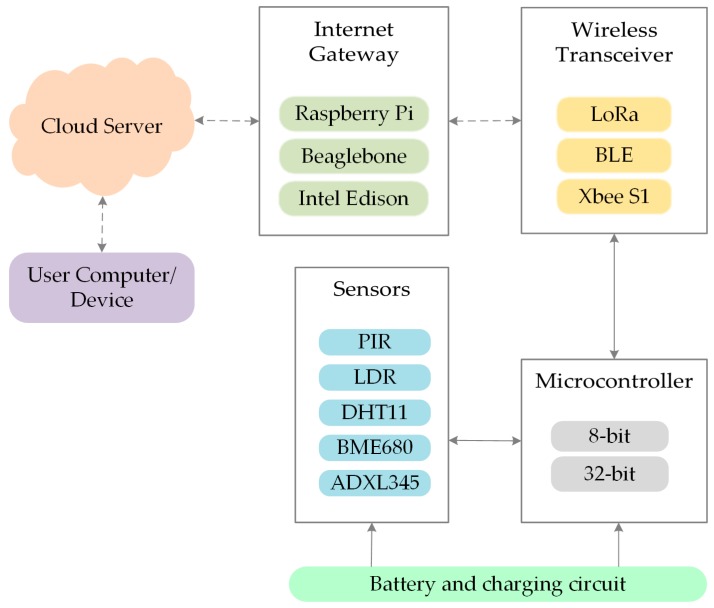
Modular LiB architecture.

The modular IoT architecture in Figure 2 consists of multiple layers namely: power source, sensors, microcontroller, wireless transceiver, internet gateway, and cloud server. The sections below provide an overview of each layer and outline the several factors to be considered while choosing among options for any IoT application.

#### 3.1.1. Power Supply

In any application, choosing an appropriate power source is essential for energy efficiency and the overall feasibility of the system. It is necessary to account for the overall power consumption and the required backup time. If the IoT system is to be placed on the human body or mounted in a remote area, it will require a battery or an energy harvester that can provide the portability. For wearable devices, batteries should not contact skin directly as the heat dissipation could cause burn [45]. Using energy harvester devices as an alternative to batteries for powering wearable devices is an emerging field of research, but such devices are still in their infancy and cannot deliver much energy [46,47]. The milliamp hours (mAh) rating is indicative of how long a battery can power a system given the current consumption rate. Although it is not enough to calculate the runtime of a system, it provides an approximation for developers to start designing the power system. The source voltage should be close to the system voltage, as there would be a waste at the conversion element if the battery voltage is higher. Linear regulators are good for stepping down voltages to system voltage and provide smooth and ripple free output at the desired voltage level, but they dissipate excess energy as heat. The drop out voltage of the linear regulator is also another parameter designers must consider while selecting components as it can be as high as two volts.

If the system voltage is too close to the battery or source voltage and the drop out voltage of the regulator is high, the regulator will not be able to provide the desired output voltage level. There are low dropout (LDO) voltage regulators that have very small dropout voltages (140 mV) and can work seamlessly with source voltages that are close to the system voltage. Linear regulators can only be used in cases where the battery or the source voltage is higher than the system voltage, and Direct Current (DC) DC–DC converters are used where the battery or the source voltage is lower than the system voltage. If a system requires linear regulators and the regulator is expected to drop significant voltage at a good amount of current, a heatsink should be mounted to dissipate the generated heat.

#### 3.1.2. Sensors

Sensors are devices that provide a quantified value of the surrounding environment such as temperature, humidity, pressure, distance, touch, etc. Sensors are the most varied element in IoT systems as different applications require different sensors. A sensor can vary from a simple limit switch to complex micromechanical accelerometers. They are used for detecting events, monitoring changes in environmental parameters and relating changes in physical characteristics to functions. Sensors are used everywhere from farms to rockets sent to outer-space. At the first layer, these sensors are interfaced to a low-power embedded processor to capture and parse raw data. As each sensor provides data in a unique format and the context of application might be different, the sensors first convert the sensed phenomena (e.g., temperature) into an equivalent electric voltage or current. A simple thermistor changes its resistivity with changes in the ambient temperature and with regression analysis, a relationship between the temperature and the corresponding resistance can be developed. Most analog sensors provide either changes in resistance or voltage to indicate changes in environmental parameters. This analog voltage is converted to a digital value by an Analog-to-Digital Converter (ADC) in microcontrollers. Digital sensors have small onboard microcontrollers and/or other circuitry built into them that provides digitized data and communicates via protocols such as Serial Peripheral interface (SPI), Inter-Integrated Circuit (I2C), Universal Asynchronous Receiver/Transmitter (UART), and Universal Serial Bus (USB).

#### 3.1.3. Microcontroller

Microcontrollers or data aggregators are essential parts that acquire the data from various sensors. They are essentially small computers with CPU, RAM, Read Only Memory (ROM), clock generation circuitry, and other functionality. Microcontrollers have peripheral features that enable them to interact and communicate with other hardware. They have output ports that can be driven high or low to control other circuitry or to initiate action in other hardware in the system. They also have pulse width modulation (PWM) enabled pins that can programmatically generate pulses to control motors, servos or in some cases provide control signals. Most microcontrollers have analog to digital converters that convert the analog values from an analog sensor into digital values to be processed and/or transmitted to the wireless transceiver. Some microcontrollers have a network stack that provides wireless connectivity to send the data to the central gateway device wirelessly. Typically, most modern microcontrollers have several power-saving modes, ideal for intermittent actions to save power.

Microcontrollers have different interrupt capabilities, including both software and hardware interrupts, so that the signal can be monitored in parallel with the main firmware, especially useful in scenarios where the reception of a control signal or span of an event is very brief. To communicate with digital sensors and other active components or transceivers, microcontrollers have an array of communication peripherals built into them such as SPI, I2C, UART, Modbus, and CAN-bus. Newer microcontrollers also have crypto-engines to make encryption faster and more power efficient in smaller devices.

#### 3.1.4. Wireless Technologies

As IoT pervades every aspect of modern life, more devices are getting connected and it is a challenge to design and support an infrastructure that can handle such dense traffic and be versatile to accommodate heterogeneous devices. Many of such edge node devices are battery powered, and restricted in terms of computational resources, memory, and transmission power. Zigbee [48,49] is a low power, low latency communication protocol conforming to IEEE 802.15.4 specifications, standardized in 2003 and is used to establish communication between battery-powered nodes in wireless sensor networks such as environmental monitoring, home automation [50], traffic monitoring, etc. Zigbee standard imposes restrictions on power usage resulting in a low data rate up to 250 kbit/s and restricts the range to 10–100 m line-of-sight. It operates in a 2.4 GHz frequency range and is ideal for low power and low bandwidth applications.

Bluetooth Low Energy (BLE) is a wireless personal area network developed by the Bluetooth Special Interest Group (SIG) and is used for short-range monitoring and control applications such as wearable electronics, medical informatics, consumer electronics, security, and entertainment systems [51]. This technology is considerably low power compared to Bluetooth Classic and BLE devices, and can run on very limited energy for extended periods of time [52]. Additionally, with majority of consumer devices having a built-in BLE transceiver, it is an ideal candidate in portable IoT applications.

LoRa (Long Range) is a wireless communication technology conforming to the IEEE 802.15.4 g protocol, developed by Semtech specifically designed for long range (up to 10 km), low power communication [53,54]. It operates in the sub-gigahertz ISM bands such as 868 MHz and 915 MHz. LoRa is the physical layer protocol and LoRaWAN is the network on which LoRa operates. Low power wide area networks (LPWANs) are cellular networks which consist of end devices and base stations forming a star-topology network. The end devices typically communicate to base stations, and one base station can facilitate thousands of end nodes.

#### 3.1.5. Internet Gateway

The Internet gateway is a device that accumulates data from all edge devices in the network and pushes it into the application management cloud server. Generally, an internet gateway device is a microprocessor that can handle large streams of data, has enough processing power to analyze the data and has internet connectivity to send the data to servers for further analysis and storage. In most cases, the internet gateway device in an IoT system is a single board computer with onboard Ethernet and/or Wi-Fi connectivity. This device is always powered on and is mains powered. Usually, data is stored locally for back-up and these data packets are sent to the server at regular intervals. This device also receives instructions from the server and configures the sensor network accordingly.

#### 3.1.6. Cloud Server

Cloud server is responsible for facilitating the end-users’ ability to access the sensed data. This is achieved by implementing several services including, but not limited to, data storage, data analytics, and data visualization in addition to providing an appropriate application program interface (API) and software tools through which the end-user can access the data. It also provides a user interface to display the data and interact with the network. Often cloud servers host an array of tools to sort and interpret the accumulated data. Security and privacy to data are one of the most common attributes to be considered in IoT applications, and accordingly, the most common platforms account for the same. Platforms such as Evrything [55], Thingworx [56], and Paraimpu [57] require an API key in the HTTP header, and an open standard for authorization. One appealing aspect of the majority of the systems is that web mashups can be created in any programming language supporting HTTP communication. Overall, each application management system has its own unique advantages and could be chosen based on the specific IoT application.

### 3.2. Encryption Algorithms

#### 3.2.1. AES Implementation

**Figure 3:**
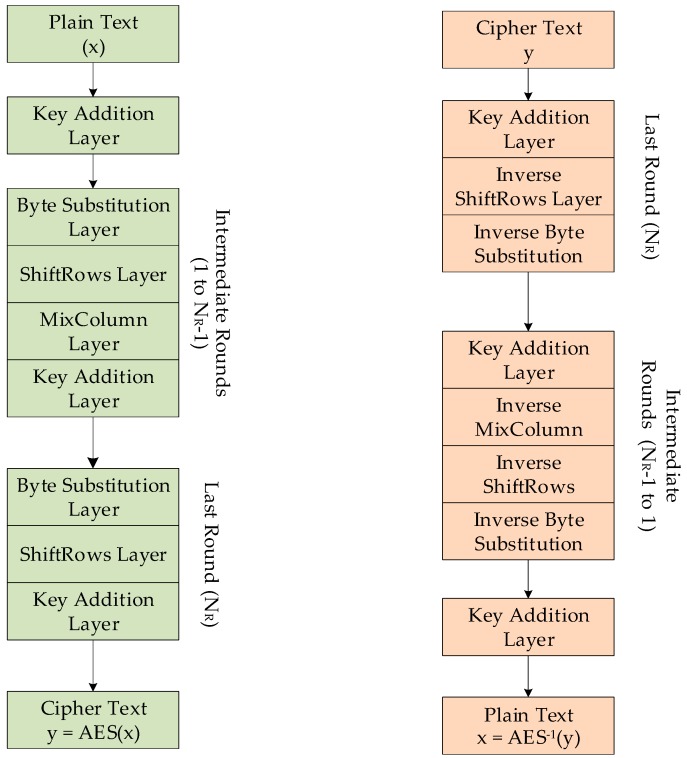
AES encryption and decryption process.

AES is a symmetric key encryption algorithm, and was originally called “Rijndael Cipher”. AES encryption converts data into ciphertext and decryption converts the ciphertext back into plaintext. The decryption algorithm differs from the encryption, although similar steps are used to encrypt and decrypt data. The encryption process can be divided into four functional blocks. AES works with 128-bit data blocks, and if the data is longer than 128 bits, it is broken up into 16 byte blocks. Each block is encrypted independently. If the message is not divisible by 16, the remaining empty bits are padded with zeros. AES requires some initialization steps and recurring rounds as in Figure 3. Initially, key expansion and the initial round are performed followed by a series of encryption rounds, using expanded keys from the key expansion step. After defined number rounds, the ciphertext is passed through one final round. The number of rounds increase (10, 12, 14) along with the key size (128, 192, 256 bits). The keys used for each round is generated from the original key in the key expansion step. So different keys generated from the original key are used for every single round.

The AES encryption process can be divided into four functional blocks, namely key expansion, initial round, recurring rounds, and final round. For 128 bit key, 44-word keys are generated in the key generation step. In case of the 192-bit key and 256-bit key, 52 and 60 words would be required, respectively. The first four words are XORed with the input matrix before the round functions begin. The remaining 40 words are used for the 10 subsequent rounds, 4 words in each round. The 128-bit key is arranged in a 4 × 4 matrix in such a way that the first 4 bytes of the key are put into the first column of the matrix. Byte-oriented operations during the encryption and decryption process are optimum for 8-bit, 16-bit, and 32-bit microcontrollers, although going from 8-bit to 32-bit architecture does not improve timing performance as evident from the results obtained in Section 5. AES-256 provides adequate security but strains system resources such as timing, memory, and energy, hindering developers from using low-power edge devices in their respective IoT applications.

#### 3.2.2. XTEA Implementation

The XTEA algorithm is the improved version of TEA, proposed by David Wheeler and Roger Needham in 1997. It is a Feistel cipher with a block size of 64 bits and a key size of 128 bits. The key schedule is simple and keys for the rounds are scheduled dynamically so it reduces the impact on memory, which is ideal for microcontrollers with low memory.

**Figure 4:**
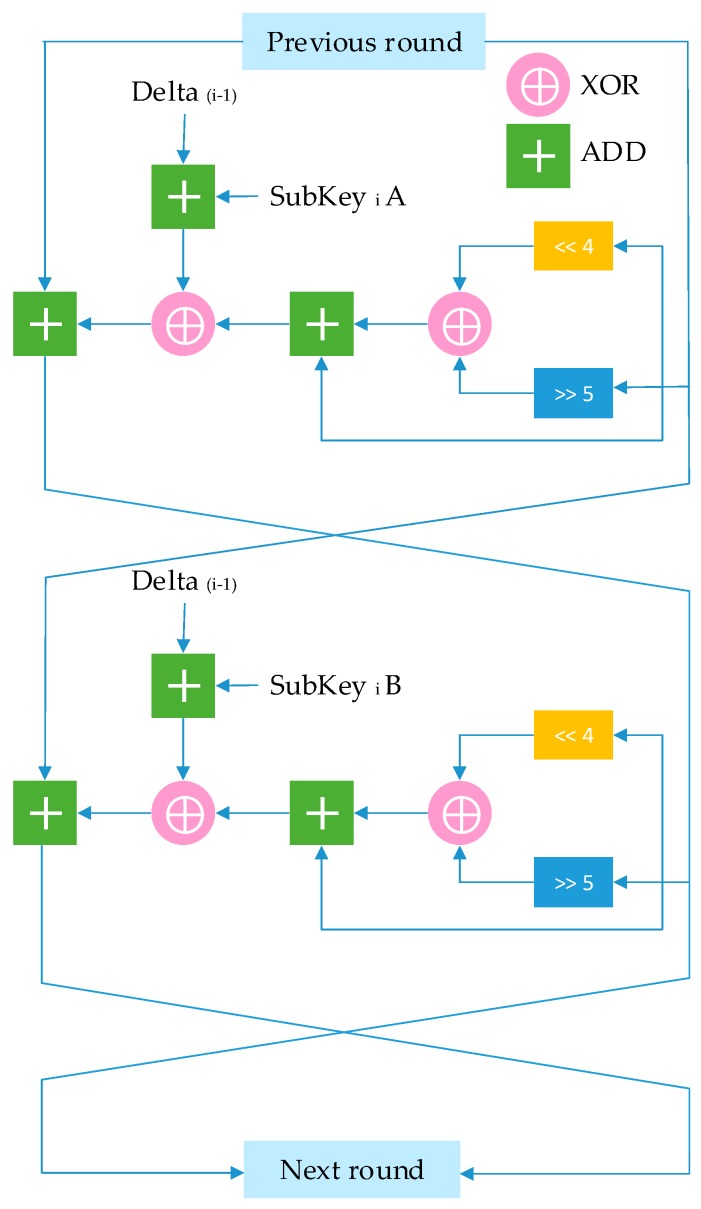
One cycle of XTEA.

Each cycle consists of two Feistel rounds. The number of rounds can be changed for different levels of security. The 128-bit key is broken down to four blocks of 32-bit subkeys. The key for each round is derived from the sum from each round. The rounds are identical and use different multiples of magic constant called delta = ($\sqrt{\text{5}}$ −1)×2^31^. The constant number ensures that the subkeys are different for each round. It requires no initialization steps and does not use S-boxes like AES which reduces memory requirement. The code consists of simple operations such as shifts, additions, and XORs. As seen in Figure 4 the data goes through alternating additions and XOR operations. Dual shift ensures the key and data are mixed properly. The 64-bit data block is split into two 32-bit variables and operations such as rotation, addition, and XOR is used to mix the data with the key for each round. The decryption inverts the operations occurred during encryption. Simple operations in encryption and decryption result in low use of RAM space and reduce overall energy consumption. Although XTEA does not provide security level on par with AES-256, its impact on computational time, memory, and energy consumption of resource constraint hardware are low, making it suitable for use in low-power, resource-constrained microcontrollers in various IoT applications where security is not too critical.

## 4. Application Implementation

Section 4.1 describes the testbed on which the encryption algorithms were implemented and evaluated on the 8-bit and 32-bit microcontrollers. Section 4.2 depicts three applications with different sensors, microcontrollers, wireless transceivers, and cloud servers. All the components are packaged in a custom 3D printed housing, making it highly portable and easy to deploy. The performance of each application was evaluated and current was measured using Tektronix DMM 4020 precision benchtop multi-meter.

### 4.1. Testbed Setup

The proposed testbed consists of two microcontrollers (8-bit and 32-bit) and their respective software and programming tools to implement the aforementioned block ciphers. The 8-bit PIC microcontroller is chosen for evaluating XTEA and software AES in low resource embedded platforms. It had 128 Kbytes [58] of program memory, sufficient to implement the software AES algorithm. This combined with eXtreme Low-Power (XLP) technology makes it energy efficient and ideal for IoT applications. As many microcontrollers for IoT edge devices such as PIC18F24K42 do not have large program memory space, software AES cannot be implemented in them [59].

**Figure 5:**
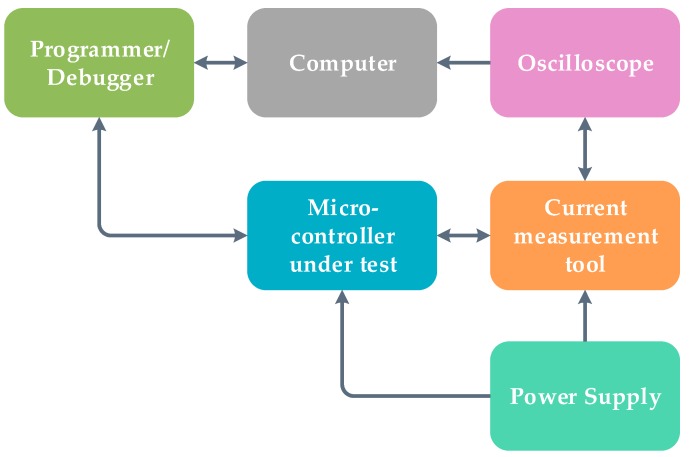
Block diagram of the testbed.

**Figure 6:**
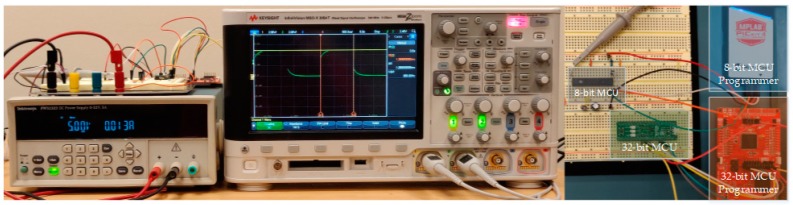
Experimental setup for measuring execution time.

The 32-bit MSP432 microcontroller has an AES hardware accelerator, enables the microcontroller to perform complex computation without consuming precious program memory, and is efficient in terms of time and power. AES can be implemented in software across different architectures (8-bit, 16-bit, and 32-bit), but is not as efficient as hardware accelerated AES. A software implementation of XTEA was done on all platforms including the 8-bit microcontroller, which does not have a hardware accelerator. From the proposed testbed, the cycles, power, code size, and RAM space required to encrypt and decrypt sample data were measured. The execution time was measured in terms of clock cycles using the oscilloscope in Figure 5 and the experimental setup in Figure 6. The internal timers inside the microcontrollers were used to record the number of cycles while encrypting and decrypting sample data. To further verify the results, the execution time was also measured with an oscilloscope.

Hardware efficiency can be measured in terms of latency, clock cycles, and power consumption. Software efficiency can be quantified by considering the number of cycles it takes to encode and decode data sets. As most sensor nodes in IoT applications have limited resources and often feed sensor data to the cloud in real time, the encryption process should not add much overhead. As most sensor nodes are either battery powered or have stringent restrictions on power, it must be ensured that data encryption does not increase the power consumption.

To measure the time required to encrypt data, one of the output pins of the microcontroller was driven high at the start of the encryption process and driven low at the end providing a pulse equal to the encryption time, which was measured with an oscilloscope. Keysight’s InfiniiVision MSO-X 3054T Oscilloscope was used to conduct all the measurements. The same process was taken to measure the timing of the decryption process. The power was measured with the help of a current measurement tool called a Real-time current monitor [60]. This tool has a precise current sensing resistor and amplification stages to measure in line low dynamic currents. The amplification stages are fast enough to capture instantaneous data which is essential as the encryption and decryption time is in the order of tens of milliseconds. The measured values were exported to the computer with the help of the USB connectivity of the current measurement tool. The instantaneous current values during encryption and decryption were multiplied with the reference power supply unit Tektronix PWS 2323 to get the power. The voltage of the power supply was measured at the device under the test end with Tektronix DMM 4020 precision benchtop multi-meter to compensate for the wire losses.

The respective timing data was used to obtain the energy used for the encryption and decryption process. The code size was acquired from the hex file generated by the compiler. The RAM space was obtained from the compiler. The optimization feature in the compilers was disabled to cancel out any interferences caused by the compiler optimization. All the microcontrollers were run at the same speed to negate the effects of the CPU clock speed on the encryption/decryption timing. The data obtained from the experiments are presented in the following sections.

### 4.2. Occupancy Monitoring

**Figure 7:**
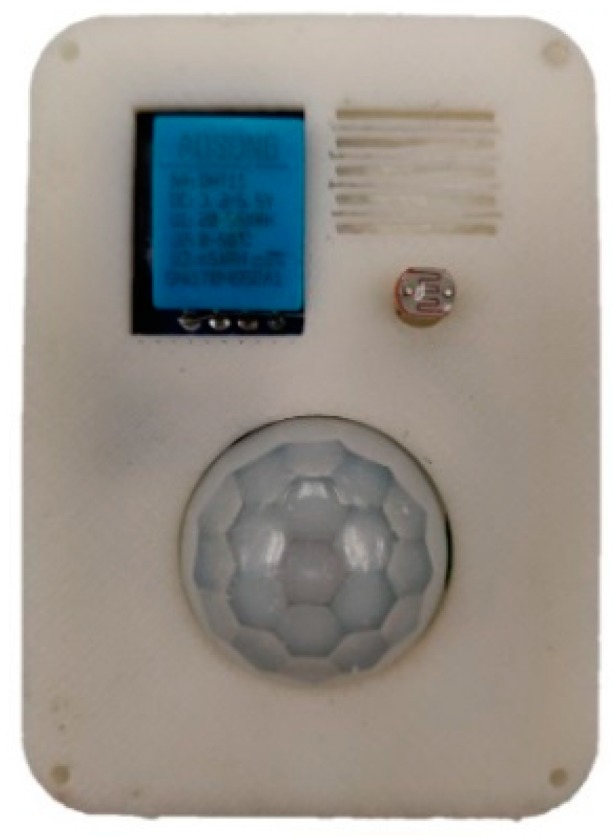
LiB prototype for occupancy monitoring.

**Figure 8:**
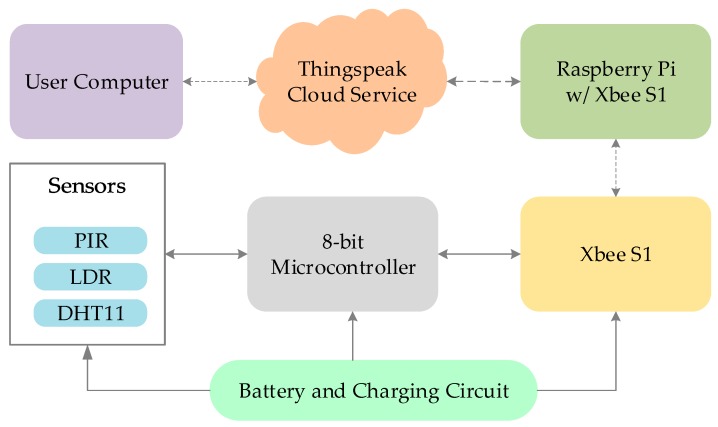
System Architecture for Occupancy Monitoring.

For indoor occupancy monitoring, the LiB IoT system was equipped with a passive infrared sensor (PIR), a light dependent resistor and a temperature and humidity sensor DHT11 and housed in a 3D printed box as in Figure 7. The ATmega8 microcontroller was used and wireless communication was achieved via a Zigbee RF module Xbee S1 [61]. All the components were chosen to keep the overall power consumption low. Figure 8 depicts the system configuration from a higher-level view. From Table 1, the total amount of current consumed by the system is 66.4 mA when the device is transmitting or in active mode, whereas during sleep state it reduced to 11.5 mA, consuming about 1/6th of the active state consumption.

DHT11 [62] stays in a low power state until communication is initiated by the microcontroller and the conversion process starts. During standby mode, the sensor only consumes around 100 µA. After the communication is initiated by the microcontroller the sensor sends a 40-bit packet containing the temperature and humidity data. The light dependent resistor changes its resistance according to the ambient light level and the analog voltage generated by the accommodating resistor divider circuit is converted to a digital signal by the 10-bit ADC in ATmega8 [63]. Without encryption, the data collected by the sensors are vulnerable to malicious intent. Attackers might monitor the occupancy in the house and find out when the house or establishment is empty and carry out crimes without any obstruction. Encrypting the data ensures that the data is safe from attacks.

A 3.7 V 200 mAh Lithium Ion battery is used to power the circuit and TP4056 charge controller is used for charging the battery. A 3.3 V low dropout linear regulator MCP1700 is used to provide a reference voltage for the ADC in the microcontroller connected to the external reference pin of the microcontroller. As the battery voltage reduces with prolonged use, the supply voltage cannot serve as accurate ADC reference voltage. Data accumulated from the sensors is sent to the internet gateway Raspberry Pi via Xbee S1 and then to Thingspeak cloud server for storage and analysis. The dashboard of the cloud service provides simple data visualization, graphs, and features such as device discovery, integration of things to the web, RESTful interaction with the platform, security, and web messaging, and tools such as MATLAB for data analytics.

### 4.3. Indoor Environmental Monitoring

**Figure 9:**
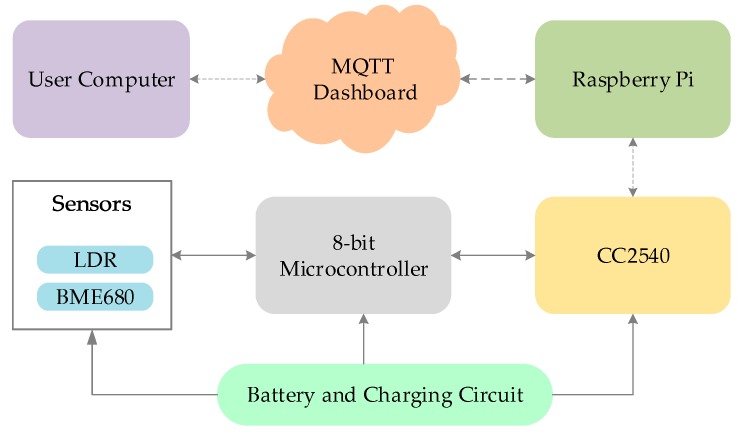
System Architecture for Indoor Environmental Monitoring.

Indoor environmental monitoring is essential to ensure a safe office and home environment. In this variant of the LiB IoT system as in Figure 9, the BME 680 sensor (Adafruit Industries, New York, NY, USA) was used to measure temperature, humidity, atmospheric pressure, and gas and a light dependent resistor was used for measuring the ambient light in rooms. The BME 680 is a low power all-in-one sensor for indoor environmental monitoring [64]. As the integrated sensor measures all three parameters, it reduces latency, power consumption, and overall efficiency of the system. The sensor communicates via SPI protocol, and can detect harmful particles in the air and gives a 0–500 point quantified value of the quality of indoor air with 1-point resolution.

The PIC18F27K40 microcontroller (Microchip Technology, Chandler, AZ, USA) had all the necessary peripherals such as SPI and ADC to collect data from the sensors and consumed low power [58]. The system is powered from a 200 mAh Li-ion rechargeable battery. From Table 2, the device consumed 0.77 mA in sleep mode and at that current, even the small 200 mAh battery can theoretically run up to 260 h. The CC2540F128 BLE system on a chip compliant with Bluetooth 4.0 technology [65] is utilized for low-power, and efficiency in integration with other devices. The internet gateway device received the BLE data and pushed it to MQTT-dashboard, where the data can be view and stored for analysis.

### 4.4. Fall Detection for Elderly

**Figure 10:**
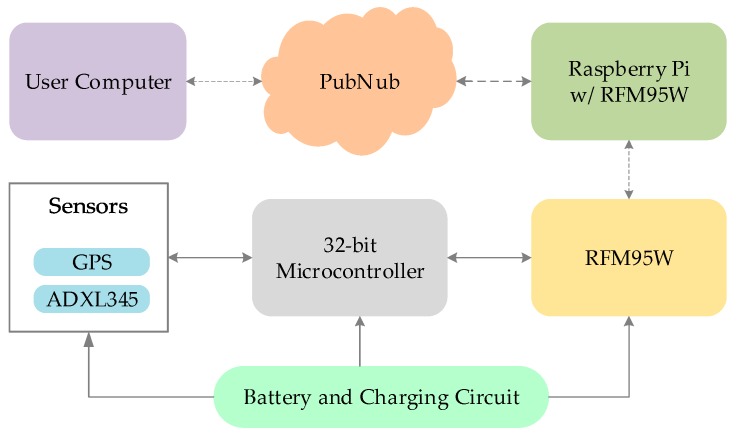
System Architecture for fall detection.

Fall detection saves the lives of many elderly and disabled people by informing relatives and emergency services. IoT system can greatly improve the health and living condition of elderly people [3]. In this variant of the LiB IoT system as in Figure 10, ADXL345 3-axis accelerometer was used to detect changes in motion to indicate a fall. It is an ultra-low power portable sensor which can also detect free fall. It can communicate both via SPI and I2C and in this case, SPI was used to integrate this sensor with MSP432 32-bit ARM microcontroller [66]. The microcontroller with low-power mode and internal real time clock was used to wake up the device during deep sleep stages and synchronize communication. MSP432 has an AES hardware accelerator, which provides security while the data is being transmitted to the LoRa device [67] without affecting the performance of the firmware [8].

As in Table 3, the majority of current is consumed by the LoRa radio RFM95W while transmitting or receiving. When a fall was detected, the accelerometer sensed a change in the X, Y, Z direction and sent it to the microcontroller, which in turn woke the LoRa radio, and transmited the information to the internet gateway, which further sent a tweet via PubNub [68]. By including a low power GPS, this system could also send location information to assist caretaker or relatives as necessary. The portability of this LiB system allows it to be strapped to the arm without any discomfort.

This is a sensitive application and the safety of the patient depends on the authentic triggering of the circuit. If the data is not encrypted, attackers can cause false alarm or disable alarms causing a detrimental effect.

## 5. Results and Discussion

The various encryption algorithms presented in Section 4 were implemented on both 8-bit and 32-bit microcontrollers to evaluate their impact of their respective performance on memory utilization, computation time, and energy consumption. The results obtained are presented in Table 4 and Table 5 for the 32-bit microcontroller and 8-bit microcontroller, respectively. As a primary focus of this research is implementing the encryption algorithm on off-the-shelf components, and identifying a solution with minimal dependence on battery power, dedicated hardware accelerators for encryption were also considered. As AES 256 hardware accelerator was available only for the 32-bit microcontroller, its performance was compared for software implementation, in addition to lightweight encryption algorithms such as XTEA and SEA in Table 4. From this data, it is evident that AES software (AES SW) implementation is by far the most resource heavy encryption in terms of execution time, memory space and energy. The hardware-accelerated AES (AES HW), on the other hand, performed most efficiently.

**Figure 11:**
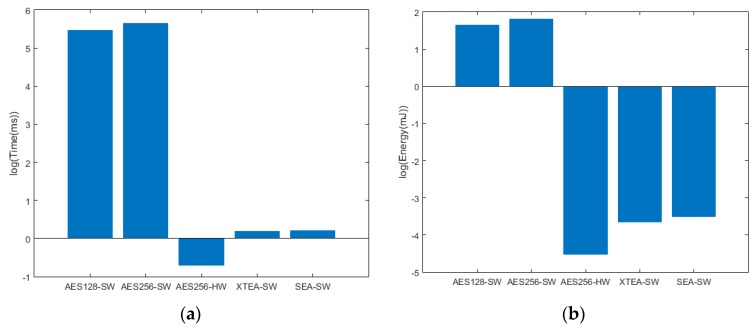
Comparative metrics for a 32-bit MCU: (**a**) Execution time; (**b**) Energy consumption.

As presented in Figure 11a, AES SW took the most amount of time (281.5 ms) as encrypting and decrypting the data requires several computationally demanding operations such as rotate, XOR, multiplication and substitution from lookup tables. The AES HW took the least amount of time (0.5 ms), over 500x less than that of the AES SW on the 32-bit microcontroller, as the dedicated and custom hardware is not restricted by the sequential completion of the CPU. The advantage of the hardware accelerator is also apparent in terms of memory. The AES SW on the 8-bit microcontroller takes 7538 bytes of code space and 1105 bytes of RAM. While designing low-power IoT systems, it would be impractical to dedicate such large amount of program memory for microcontrollers as they are already severely constrained, whereby many 8-bit microcontrollers do not even have the memory capacity for AES to be implemented in the software.

**Figure 12:**
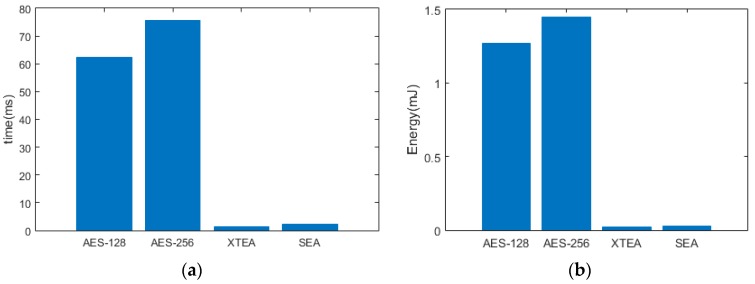
Comparative metrics for an 8-bit MCU: (**a**) Execution time; (**b**) Energy consumption.

Accordingly, alternative lightweight encryption algorithms such as XTEA and/or SEA could be utilized at the expense of a slight reduction in security. Results in Table 4 and Figure 11 show that XTEA and SEA require only 556 and 983 bytes of memory, while consuming only 0.026 and 0.03 mJ of energy, respectively, highlighting their efficiency in comparison with AES SW implementations. As the 8-bit microcontroller does not have the AES HW accelerator, AES SW was implemented and found to take 60x more cycles than XTEA as in Figure 12a. This is also reflected in Figure 12b where the AES SW consumed 60x more energy than XTEA and 48x more energy than SEA. This clearly demonstrates that light weight encryption algorithms are much more practical in 8-bit microprocessor based IoT applications.

As seen from Table 4 and Table 5, XTEA requires less code space and RAM than AES SW and is almost on par with AES HW in terms of execution time and energy. While it is clear from the evaluations that AES HW was most efficient, majority 8-bit microcontrollers that are implemented in low-power IoT applications do not have this peripheral feature. As timing and energy performance of XTEA is close to that of hardware accelerated AES, it would be practical to implement XTEA on resource constraint IoT systems, with the understanding that data is encrypted at a basic level unlike with the AES algorithm.

**Figure 13:**
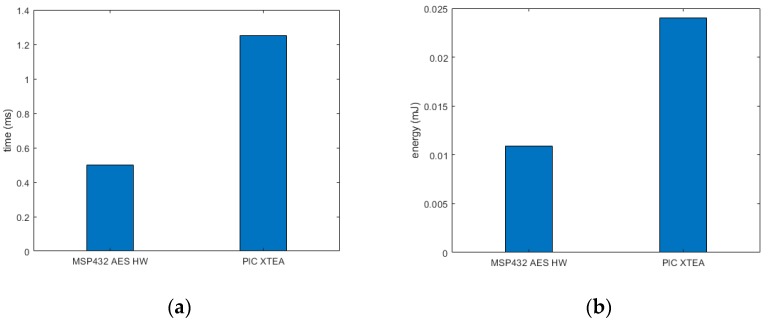
Comparative metrics for different encryption algorithms: (**a**) Execution time; (**b**) Energy consumption.

Figure 13a,b presents the comparison of AES implemented in 32-bit MCU with a hardware accelerator and XTEA implemented on an 8-bit microcontroller. Although the XTEA took longer to execute and as a result spent more energy, it is very close to the execution time and consumed energy by the 32-bit MCU while running hardware accelerated AES. Therefore, AES-256 is suitable for applications that handle very sensitive data and the need for security is highly critical. AES-256 can be employed in devices that have hardware accelerators or memory large enough so that the software implementation leaves enough room for the application firmware.

Optimizing the security required by a specific IoT application within constrained resources is a challenge as one needs to consider the temporal relevance of the data being protected for the encryption algorithm to be feasible in an application. After obtaining insight from the performance evaluation of encryption algorithms, their impact on performance of three applications in Section 4 was tested as in Table 6. Due to security risks involved when raw data is transmitted from the wireless transceiver layer to the internet gateway layer, the data needs to be encrypted prior to being transmitted to the wireless transceiver. This ensures that privacy and security are not sacrificed even if the wireless transceiver does not have any built-in security features. Continuing with the modular architecture of the LiB system, security was added by encrypting the sensor data with XTEA and AES algorithms. Comparisons in Table 6 indicate a very small increase in energy consumption as each byte read from the sensors is encrypted and transmitted to the central gateway, which then submits the data to the cloud services.

Although the energy consumption has not been affected significantly by the addition of encryption, the delay and overhead in data introduced during the encryption stage results in additional energy consumption and can deplete batteries sooner than expected. Software implementation of AES-256 consumed the most energy, followed by XTEA, in occupancy monitoring LiB and indoor environmental LiB. Finally, the hardware accelerated AES-256 consumed the least energy as seen from the data obtained from fall detection LiB.

**Figure 14:**
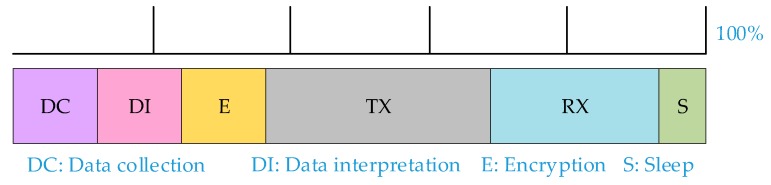
Average energy distribution chart of three applications.

Figure 14 depicts the average energy distribution graph for the three IoT applications. The energy distribution graph spans from data collection from the sensor, data interpretation and encryption at the microcontroller, sending the data packet through the wireless transceiver to the gateway, waiting for an acknowledgement receipt, and going to sleep. It is evident that most energy is consumed during transmission and acknowledgement of receipt from the internet gateway. Adding data encryption either using dedicated hardware accelerators or light weight algorithms had minimal impact on the overall energy consumption. Overall, while encryption does introduce a small delay and consumes energy, it is justified from the benefits of security and privacy.

As more heterogeneous devices are getting connected to the internet for various services, interoperability decreases because of ad-hoc networks, hardware specific solutions and lack of cloud services to cover the extent of IoT applications. When designing an IoT system, developers should consider modularity and extensibility of the system. This paper presents three low power battery powered IoT applications in portable housing for maximum portability with different hardware and cloud services. As seen from the experimental results from Table 1, Table 2 and Table 3, and Figure 14, it is evident that transmitting data requires most energy so transmission periods should also be minimized. Sensors and microcontrollers that can go into sleep states are preferred as it reduces power consumption and avoids energy wastage during idle periods. Lightweight encryption algorithms in IoT applications provide necessary security and ensures privacy at all layers, all the while introducing a small delay and energy consumption. LiB provides a modular architecture based on which different platforms and heterogeneous components can be integrated to provide a lightweight, highly portable, and secure sensor platform. Overall, while numerous studies have been published on simulation-based work, there is a lack of published literature on real-time measurements of memory, timing, power, and energy in IoT architecture implementation. This research hopes to fill this gap and provide designers a better estimation of these real-time measurements in their respective future implementations [69].

## 6. Conclusions

Integrating heterogeneous IoT systems towards a modular architecture is a challenge. While there are many application-specific solutions for environmental monitoring, the majority are not integration friendly and often result in high deployment time. The broad nature of applications require a modular architecture that can be rapidly deployed. The proposed LiB IoT architecture provides a sensor hub for different environmental monitoring applications, classifies the IoT structure into functional layers and provides simple means of modularity between the layers. This research proposes a layer of protection between a wireless transceiver and internet gateway device against remote attacks by employing lightweight encryption algorithms and evaluates the impact on timing and energy consumption.

Utilizing open-source off-the-shelf components and widely accepted encryption algorithms, this research presented a comparative study of multiple encryption algorithms to analyze the performance in memory, energy, and execution time. From the experimental evaluations, this research shows that energy efficiency and execution time of XTEA on a microcontroller without a dedicated AES hardware accelerator is almost on par with that of a device which has a crypto engine. Additionally, XTEA was around 60x more efficient in terms of power compared to a software implementation of AES on an 8-bit microcontroller while only taking 1/6th of the program memory. From analyzing the performance of AES and XTEA on different architectures, it is clear that the encryption algorithm needs to be considered closely along with the IoT system architecture.

Edge devices with abundant computational resources can implement AES in software with room for application firmware. However, as the majority of IoT applications utilize low-power edge devices, implementing AES becomes infeasible. Additionally, while a few 16-bit and 32-bit edge devices have dedicated hardware accelerators, the majority of 8-bit edge devices do not offer this peripheral feature. Without any encryption scheme, these edge devices are susceptible to malicious attacks. Accordingly, light weight encryption algorithms such as XTEA or SEA would be an ideal alternative to obtain an acceptable level of security, while at the same time reduce the deployment time. The efficiency of these algorithms has been evaluated through implementation on several IoT applications. Overall, the proposed LiB architecture takes a modular approach of designing IoT applications to address the challenges that lie in deploying a platform independent, scalable, secure and interoperable system.

## Figures and Tables

**Table 1:** Current consumption in the occupancy monitoring system.

Component	Active Current (mA)	Sleep Current (mA)
8-bit MCU	5	1
XBee S1	50	0.03
PIR	10	10
LDR	0.4	0.4
DHT11	1	0.1
**Total**	66.4	11.5

**Table 2:** Current consumption in indoor environmental monitoring system.

Component	Active Current (mA)	Sleep Current (mA)
8-bit MCU	0.2	0.06
CC2540 BLE	30	0.3
BME680	0.1	0.01
LDR	0.4	0.4
**Total**	30.7	0.77

**Table 3:** Current consumption in fall detection system.

Component	Active Current (mA)	Sleep Current (mA)
32-bit MCU	4	0.8
RFM95W	50	0.8
ADXL345	0.2	0.01
**Total**	54.2	1.6

**Table 4:** Performance comparisons of encryption algorithms on 32-bit microcontrollers.

	AES-128	AES-256		XTEA	SEA
		SW	HW		
**Size (bytes)**	13609	16251	1258	1946	3765
**RAM (bytes)**	2956	3508	576	556	983
**Time (ms)**	237.3	281.5	0.5	1.196	1.23
**Energy (mJ)**	5.2	6.1	0.0109	0.026	0.03

**Table 5:** Performance comparisons of encryption algorithms on 8-bit microcontrollers.

	AES-128	AES-256	XTEA	SEA
**Size (bytes)**	6368	7538	1184	2296
**RAM (bytes)**	986	1105	99	126
**Time (ms)**	62.3	75.62	1.25	2.3
**Energy (mJ)**	1.27	1.45	0.024	0.03

**Table 6:** Energy consumption of IoT applications with and without encryption for single data read and transmission.

Application	Without Encryption	With Encryption	
		AES-256	XTEA
Occupancy Monitoring System	357.46	382.89	378.76
Indoor Environmental Monitoring	167.13	183.53	176.39
Fall Detection for Elderly	182.02	186.43	203.41
